# Postnatal surgery for myelomeningocele in neonates: neurodevelopmental outcomes

**DOI:** 10.55730/1300-0144.5561

**Published:** 2022-11-30

**Authors:** Melek AKAR, Gülşah ÇETİN, Mine İNAL AKKAYA, Mehmet HELVACI, Nihal OLGAÇ DÜNDAR, Fırat ERGİN, Özgün UYGUR, Sinem AKBAY, Nail ÖZDEMİR, Meltem KOYUNCU ARSLAN, Mehmet Yekta ÖNCEL

**Affiliations:** 1 Division of Neonatology, İzmir Tepecik Training and Research Hospital, University of Health Sciences, İzmir, Turkey; 2 Department of Pediatrics, Division of Neonatology, İzmir Faculty of Medicine, University of Health Sciences, İzmir, Turkey; 3 Division of Child Development, İzmir Tepecik Training and Research Hospital, University of Health Sciences, İzmir, Turkey; 4 Division of Pediatrics, İzmir Tepecik Training and Research Hospital, University of Health Sciences, İzmir, Turkey; 5 Division of Pediatrics, İzmir Faculty of Medicine, University of Health Sciences, İzmir, Turkey; 6 Department of Pediatrics, Division of Pediatric Neurology, İzmir Kâtip Çelebi University, İzmir, Turkey; 7 Department of Neurosurgery, İzmir Tepecik Training and Research Hospital, University of Health Sciences, İzmir, Turkey; 8 Department of Pediatrics, Division of Neonatology, İzmir Kâtip Çelebi University, İzmir, Turkey

**Keywords:** Myelomeningocele, neurodevelopment, newborn

## Abstract

**Background/aim:**

The study aims to evaluate the neurodevelopmental outcomes of neonates with myelomeningocele (MMC) operated in the postnatal period.

**Materials and methods:**

This is a prospective follow-up study in a tertiary neonatal intensive care unit. Neurodevelopmental outcomes of term neonates operated for MMC and healthy term newborns were compared with the Bayley Scales of Infant and Toddler Development-Third Edition (BSID III) at 12–18 months.

**Results:**

A total of 57 cases were included in the study (patient group = 27; control group = 30). Demographic data between the groups were similar. Cognitive, linguistic, and motor composite scores of the patient group were lower than those of the control group (p < 0.001). In the patient group, those who underwent ventriculoperitoneal shunt had lower cognitive, language and motor scores than those without shunt (p < 0.05). The cognitive, linguistic, and motor composite scores in the patient group who underwent surgery before 72 h were better than those who underwent surgery after 72 h.

**Conclusion:**

In our study, it was found that the neurodevelopmental prognosis of MMC cases requiring ventriculoperitoneal shunt in the postnatal period was significantly worse than those without shunt. It is the first study in which the neurodevelopment of patients with MMC who were operated in the postnatal period was evaluated with BSID III evaluated and delays in all areas were shown in cases with MMC compared to normal cases. Better neurodevelopmental outcomes in patients operated in the first 72 h suggest that early surgery will improve neurodevelopmental outcomes in patients with MMC.

## 1. Introduction

Neural tube defects (NTD) are the most common congenital malformations after congenital heart defects and occur due to the failure of the neural tube to close spontaneously, which should be closed by the third to fourth week in intrauterine life. Myelomeningocele (MMC) is the most common NTD, characterized by protrusion of the meninges and spinal cord through open vertebral arches leading to paralysis [1].

With nearly 60 cases per 100,000 births in the United States, the prevalence of MMC varies among countries. MMCs may be part of a genetic syndrome or may occur as an isolated defect. With both environmental and genetic factors affecting the emergence of the defect, the causes remain uncertain. It has been reported that many factors may lead to the development of MMC including maternal exposure to hyperthermia during pregnancy, use of alcohol, valproic acid, carbamazepine or isotretinoin, maternal malnutrition or obesity, low folic acid levels, maternal diabetes, and genetic markers (mutations in folic acid-responsive or folic-acid-dependent enzyme pathways) [2,3,4].

A surgical correction should be performed as soon as possible following the diagnosis of neural tube defects. However, since other system disorders such as mental and motor retardation, bowel and bladder dysfunction, orthopedic problems can accompany NTDs in varying degrees, the treatment applied may bring limited success. Patients suffering from NTD often require long-term care, leading to a significant financial burden on healthcare costs [5].

Today, NTD patients who lost their lives in childhood in the past can reach adulthood to a large extent. A close follow-up with a multidisciplinary approach is required from birth to preserve the neurological functions of these patients as much as possible and to overcome orthopedic and urinary complications as they reach adulthood. It is of great importance to follow the neurodevelopment of patients and to support them with appropriate methods when needed in terms of their neurological prognosis. However, unfortunately, the literature contains very few studies evaluating the neurodevelopmental outcomes of cases with MMC and investigating the factors that may positively affect these results.

The current study aims to determine the demographic data of term newborns who were operated in the postnatal period with the diagnosis of MMC in the neonatal intensive care unit of our hospital and to evaluate the neurodevelopmental results with BSID III.

## 2. Materials and methods

### 2.1. Ethical considerations

This is a prospective follow-up study. The study protocol was approved by the local committee (Committee approval date: 21/07/2017; number: 10).

### 2.2. Study design

The cases included in the study were divided into two groups:

***Patient group (group 1):*** The patient group includes term newborn babies who were operated in the postnatal period with the diagnosis of MMC in the neonatal intensive care unit of our hospital between January 2018 and December 2019.

***Control group (group 2):*** The control group includes healthy term newborn babies who applied to the pediatric outpatient clinics for different reasons.

***Criteria for exclusion from the study:***

Premature cases (week of gestation <37 weeks), cases with multiple organ anomalies, cases diagnosed with metabolic disease or hypoxic ischemic encephalopathy, and with low birth weight according to gestational week or with intracranial bleeding were excluded from the study.

Demographic information and clinical characteristics of the patients in the patient and control groups were compiled retrospectively from their hospital records.

Neurodevelopmental evaluations including cognitive, language, motor, social-sensory and adaptation behaviors of both the patients in the patient group and the control group were performed by a certified child development specialist with the BSID III at 12–18 months.

### 2.3. Statistical analysis

In this study, all data were analyzed with the SPSS 20.0 program. Numerical data were expressed as arithmetic mean ± standard deviation (min-max) or median, and categorical data were expressed as numbers and percentages, depending on whether they showed parametric properties or not. Student-t, Mann-Whitney U and chi-square tests were used to compare two independent groups. A p value of <0.05 was considered statistically significant.

## 3. Results

A total of 57 cases, 27 in the patient group and 30 in the control group, were included in the study. Demographic information of both groups is shown in Table 1. The patient group and control group were found to be similar in terms of demographic data (p > 0.05).

When the mothers of the cases in Group 1 were evaluated in terms of neural tube defect etiology, it was seen that there were no mothers who were exposed to the drug or another teratogen, except for two mothers who used valproic acid due to epilepsy and were learned that they did not take folic acid supplementation during their pregnancies. Mothers of the cases in group 2 did not have radiation, hyperthermia, and drug exposure during pregnancy.

It was seen that 81% of the cases in group 1 had a prenatal diagnosis and three of them had NTD individuals in their families. The other cases in this group had no prenatal diagnosis.

Distribution of cases according to MMC localization and accompanying anomalies is shown in Table 2.

Epilepsy developed in 3 (11.1%) of the patients in grup 1 during the follow-up and antiepileptic treatment was started.

It was seen that 55% of the cases in group 1 were operated within the postnatal 72 h and the patients were operated on the mean postnatal 4th ± 3.54 day (1–13 days).

It was determined that ventriculoperitoneal (V/P) shunt was placed in thirteen of the cases in group 1, shunt revision was required in four cases, and shunt infection developed in three of the cases that underwent shunt revision. Ventriculitis was not observed in any patient. While the cases in group 1 were followed up in the neonatal intensive care unit, clinical sepsis developed in nine, proven sepsis in four, and meningitis in one.

Bayley Scales of Infant and Toddler Development-Third Edition test application age was 16.07 ± 5.53 months in group 1 and 17.4 ± 4.43 months in the control group. The two groups were found to be similar in terms of test administration time (p = 0.326).

Table 3 shows the Bayley cognitive scale score, Bayley language scale score, Bayley motor scale score, and Bayley composite score distributions of both groups. Cognitive scale score examined with BSID III, receptive, expressive, and total language scale scores, fine, gross, and total motor scale scores, and cognitive composite scores were found to be statistically significantly lower in the cases in group 1 compared to the cases in group 2.

In the study, the cases in group 1 were divided into two subgroups as cases with V/P shunts (group 1a) and cases without (group 1b) to determine the factors that negatively affect their neurodevelopment. While the number of cases was 13 in group 1a, it was 14 in group 1b.

The distribution of Bayley cognitive scale score, Bayley language scale score, Bayley motor scale score and Bayley composite score of the cases in groups 1a and 1b are shown in Table 4. Cognitive scale score measured with BSID III, receptive and total language scale scores were found to be fine, and total motor scale scores were found to be lower in Group 1a compared to group 1b patients (p < 0.05). The expressive language scale score was 3.23 ± 2.86 (1–11) in group 1a and 7.07 ± 6.15 (2–18) in group 1b (p = 0.051), while the gross motor scale score was 3.00 ± 2.79 (1–11) in group 1a and 6.79 ± 6.21 (1–17) in group 1b.

The Bayley cognitive composite score (p = 0.004) and Bayley total language composite score were found to be lower in the subjects in group 1a compared to the subjects in group 1b (p = 0.001). Bayley motor composite score was found to be lower in the cases in group 1a compared to the cases in group 1b (p < 0.001).

In addition, the cases in group 1 were divided into two subgroups as those who had surgery before the postnatal 72 h and those who did not. When both groups were evaluated with BSID III, the cognitive, language and motor composite scores of the patients who had surgery before the postnatal 72 h were better than those who had surgery after 72 h, but there was no statistically significant difference between them (p > 0.05).

## 4. Discussion

Myelomeningocele is a common congenital malformation that occurs in the first month of embryological life and often causes permanent deformities throughout life. Despite the improvement of antenatal diagnosis possibilities and the increasing effectiveness of folic acid as a preventive factor, MMC continues to be an important health problem in our country and in the world [6,7].

There is not yet a consensus in the literature about how the delivery method of myelomeningocele cases should be. In some studies in the literature, it is accepted that delivery by normal spontaneous vaginal route causes spinal cord injury and poses a risk factor for a poor neurological condition, while other studies report that the mode of delivery does not affect the neurological prognosis [8]. In the current study, it was determined that most of the patients (81.5%) were delivered by C/S. The reason for this may be that there is no consensus yet in our country about the mode of delivery in cases with MMC and the high rate of C/S delivery in our country.

It has been reported that the management of the delivery room of babies with neural tube defects is important for the neurological prognosis of the cases. Especially, cases with MMC who do not have an intact skin tissue should be laid on their side immediately after birth, care should be taken not to tear the sac, and the defect should be covered with gauze moistened with saline [9]. All the patients in the current study were delivered in the delivery room of our own hospital and were managed in accordance with the guidelines in the delivery room.

It was thought that correction surgeries to be performed in the prenatal period in myelomeningocele cases would positively affect the neurodevelopmental prognosis. However, in the literature, prematurity is seen more frequently in MMC cases who were operated on prenatally, but neurodevelopmental damage is reported to be similar when compared with cases operated postnatally [10]. All of the cases in this study were operated in the postnatal period.

In the literature, it has been reported that cases with MMC have a high risk of cognitive dysfunction, which becomes especially evident with increasing age. Studies show that verbal and performance IQ scores and arithmetic and reading skills of children with MMC are lower than those of children without MMC [11–13]. However, there are very few studies in the literature evaluating the neurodevelopmental outcomes of infants with MMC. In the study by Johnson et al., 51 fetuses with MMC who were operated during the fetal period were evaluated with BSID II when they reached the postnatal two years of age and cognitive language and personal-social skills were within the normal range in 67% of the cases, while mild delay was observed in 20% and significant delay in 13% [5]. To the best of our knowledge, this is the first study to evaluate the neurodevelopment of patients with MMC who were operated in the postnatal period with BSID III in the period of 12–18 months. In the current study, MMC cases operated in the postnatal period were compared with the control group and supporting the studies in the literature, the cognitive scale score found with BSID III recipient, expressive and total language scale scores, fine, gross and total motor scale scores and cognitive composite scores were found to be statistically significantly lower in patients with MMC than in the control group.

Although it has been reported in the past that the presence of hydrocephalus and V/P shunts in patients with MMC cause significantly lower scores in neurodevelopmental and cognitive tests, today, it is accepted that the presence or absence of shunt does not affect neurodevelopmental tests unless there are additional problems such as central nervous system infection or intracranial bleeding [14–17]. In the current study, the cognitive, language and motor composite scores of the cases with V/P shunts were found to be statistically significantly lower than those without. In the study, shunt revision required due to shunt infection in four cases and shunt infection developed in three of them, but ventriculitis was not observed in any case. Due to the low number of cases, infection-related neurodevelopmental outcomes in patients with shunt could not be evaluated. According to the results of our study, MMC cases with V/P shunts should be monitored more closely, should be supported in terms of neurodevelopmental problems and close cooperation should be made with families.

Due to the high risk of meningitis in myelomeningocele cases, prophylactic antibiotics are recommended in patients until the defect is closed with surgery. When antibiotic prophylaxis was compared in MMC cases, it was reported that the rate of ventriculitis development was higher in those who were not given antibiotics [18]. In the current study, broad-spectrum antibiotics (ampicillin and cefotaxime) were started in all cases with MMC that were not covered by an intact skin until the defect was closed, and after the defect was closed, it was decided to discontinue antibiotics according to the clinical and laboratory results of the patients.

It has been reported that early and aggressive surgical intervention is associated with low morbidity and mortality in cases with myelomeningocele. In a study by Öncel et al., including 30 MMC cases, early surgical intervention (≤5 days), short hospital stay, and antibiotic therapy were associated with a low complication rate [19]. In another study by Bülbül et al., it was determined that the length of stay, duration of antibiotic administration, and early complications were significantly lower in babies who underwent correction surgery in the first 3 days of life [20]. In the current study, the mean duration of taking antibiotics for the patients who underwent surgery within 72 h was 16.2 ± 5.4 days, while it was found to be 18.08 ± 9.53 days shorter than the mean duration of antibiotics for those who underwent surgery after 72 h, however, no statistically significant difference was found between the two groups.

To the best of our knowledge, there is no study in the literature comparing the effects of early or late surgery on the neurodevelopment of MMC cases in the postnatal period with the BSID III. In the current study, although the cognitive, language and motor composite scores of the patients who underwent surgery before 72 h were better than those who underwent surgery after 72 h, no statistically significant difference was found between them. Due to the small number of cases in our study, we recommend to support our outcomes by studies with a higher number of cases investigating the effect of early/late surgical intervention on neurodevelopmental outcomes.

In this study, it was found that the neurodevelopmental prognosis of MMC cases requiring ventriculoperitoneal shunt in the postnatal period was significantly worse than those without shunt. It is the first study in which the neurodevelopment of patients with MMC who were operated in the postnatal period was evaluated with BSID III and delays in all areas were shown in cases with MMC compared to normal cases. Better neurodevelopmental outcomes in patients operated in the first 72 h suggest that early surgery will positively change neurodevelopmental outcomes in patients with MMC.

Detection of problems at an early age with early and regular follow-up in MMC cases increases the quality of life in these babies. Close follow-up of these patients with developmental tests and after surgery, evaluation and treatment of the problems positively affect the neurodevelopmental prognosis of these patients.

## Figures and Tables

**Table 1:** Demographic characteristics of the groups.

	*Group 1 (n = 27)*	*Group 2 (n = 30)*	*p*
Maternal age, year*	27.11 ± 5.97	26.6 ± 4.9	0.893
Gestational week*	38.18 ± 1.46	38.23 ± 1.16	0.892
Birth weight, g*	3118.14 ± 407	4139.66 ± 530	0.302
Head circumference, cm*	35.40 ± 3.0	35.40 ± 1.4	0.991
Male sex (%)	9 (33)	15 (50)	0.432
Cesarean section (%)	22 (81)	25 (83)	0.826
Folic acid supplementation (%)	9 (33)	24 (80)	**<0.001**

* As presented mean ± SD.

**Table 2:** Localization of myelomeningocele and additional anomalies.

Localization of myelomeningocele	n (%)
Thoracolumbosacral	1 (3.7)
Thoracic	2 (7.4)
Cervikal	2 (7.4)
Lumbosacral	3 (11.1)
Sacral	5 (18.5)
Thoracolumbar	6 (22.2)
Lumbar	8 (29.6)
**Additional anomalies**	**n (%)**
Arnold-Chiari malformation (type 2)	13 (48.1)
Pes equinovarus	10 (37)
Hydrocephalus	7 (25.9)
Secundum atrioventricular septal defect	2 (7.4)
Ventricular septal defect	2 (7.4)
Patent foramen ovale	1 (3.7)
Horseshoe kidney	1 (3.7)

**Table 3:** Distribution of Bayley scores of the groups.

	Group 1		Group 2		*p*
	Mean ± SD	Min-Max	Mean ± SD	Min-Max	
**Cognitive scale**	5.44 ± 5.50	1–18	10.43 ± 0.77	10–12	**<0.001**
**Recipient language**	5.15 ± 5.33	1–18	10.57 ± 0.77	10–12	**<0.001**
**Expressive language**	5.22 ± 5.15	1–18	10.57 ± 0.77	10–12	**<0.001**
**Total language**	10.37 ± 10.43	2–36	21.13 ± 1.54	20–24	**<0.001**
**Fine motor**	5.11 ± 5.22	1–18	10.10 ± 0.30	10–11	**<0.001**
**Gross motor**	4.96 ± 5.16	1–17	10.23 ± 0.43	10–11	**<0.001**
**Total motor**	73.44 ± 29.07	2–35	20.33 ± 0.66	20–22	**<0.001**
**Cognitive compound score**	73.44 ± 29.07	13–40	102.17 ± 3.86	100–110	**<0.001**
**Total language compound score**	71.52 ± 30.79	47–147	103.4 ± 4.64	100–112	**<0.001**
**Motor compound score**	70.22 ± 31.1	46–145	101.1 ± 2.24	100–107	**<0.001**

**Table 4:** Distribution of Bayley scores according to the presence of shunt in group 1.

	Group 1a		Group 1b		*p*
	Mean ± SD	Min-Max	Mean ± SD	Min-Max	
**Cognitive scale**	2.85 ± 2.79	1–11	7.86 ± 6.34	1–18	<0.05
**Recipient language**	2.77 ± 2.55	1–10	7.36 ± 6.32	1–18	<0.05
**Expressive language**	3.23 ± 2.86	1–11	7.07 ± 6.15	2–18	0.051
**Total language**	6.00 ± 5.29	2–21	14.43 ± 12.44	3–36	<0.05
**Fine motor**	3.00 ± 2.51	1–10	7.07 ± 6.33	1–18	<0.05
**Gross motor**	3.00 ± 2.79	1–11	6.79 ± 6.21	1–17	0.055
**Total motor**	6.00 ± 5.30	2–21	13.86 ± 12.53	2–35	<0.05
**Cognitive compound score**	64.23 ± 13.97	55–105	82 ± 36.69	13–140	0.004
**Total language compound score**	58.62 ± 15.69	47–103	83.50 ± 36.71	47–147	0.001
**Motor compound score**	58 ± 15.92	46–103	81.57 ± 37.59	46–145	<0.001
